# Multi-QTL Mapping for Quantitative Traits Using Epistatic Distorted Markers

**DOI:** 10.1371/journal.pone.0068510

**Published:** 2013-07-09

**Authors:** Shang-Qian Xie, Jia Wen, Yuan-Ming Zhang

**Affiliations:** Statistical Genomics Group, State Key Laboratory of Crop Genetics and Germplasm Enhancement, Department of Crop Genetics and Breeding, Nanjing Agricultural University, Nanjing, Jiangsu, China; Nanjing Forestry University, China

## Abstract

The interaction between segregation distortion loci (SDL) has been often observed in all kinds of mapping populations. However, little has been known about the effect of epistatic SDL on quantitative trait locus (QTL) mapping. Here we proposed a multi-QTL mapping approach using epistatic distorted markers. Using the corrected linkage groups, epistatic SDL was identified. Then, these SDL parameters were used to correct the conditional probabilities of QTL genotypes, and these corrections were further incorporated into the new QTL mapping approach. Finally, a set of simulated datasets and a real data in 304 mouse F_2_ individuals were used to validate the new method. As compared with the old method, the new one corrects genetic distance between distorted markers, and considers epistasis between two linked SDL. As a result, the power in the detection of QTL is higher for the new method than for the old one, and significant differences for estimates of QTL parameters between the two methods were observed, except for QTL position. Among two QTL for mouse weight, one significant difference for QTL additive effect between the above two methods was observed, because epistatic SDL between markers C66 and T93 exists (*P* = 2.94e-4).

## Introduction

Quantitative trait locus (QTL) mapping has become a routine approach in genetic studies of quantitative traits [1]–[5]. Most QTL mapping approaches usually make use of markers that follow a Mendelian segregation ratio. However, some markers often show distorted segregation ratios from Mendelian expectations in actual QTL mapping populations [6]–[8]. This segregation distortion often affects linkage group construction and QTL mapping results [9]–[11]. Therefore, how to use distorted markers in QTL mapping needs to be addressed.

Segregation distortion is a commonly encountered phenomenon [12]. Several mechanisms or approaches have been proposed to explain this phenomenon [10], [13]. Previous studies on the influence of distorted markers mainly focus on two aspects. First, this distortion may lead to biased estimate of recombination fraction between distorted markers. To solve this issue, Lorieux et al. [14]–[15] adopted two-point method to correct genetic distance between distorted markers. Zhu et al [9] extended multipoint analysis method to more general situations, considering distorted, dominant and missing markers at the same time. Recently, Xie [11] considers epistasis between two linked SDL in the construction of linkage groups. Second, this distortion affects QTL mapping results, for example, QTL detection power [16]–[17] and QTL additive effect [10]. To improve the precision of QTL mapping, Xu and Hu [18] developed an interval mapping approach for joint analysis of both QTL and SDL. Recently, Wen et al. [10] further extended a multi-QTL mapping approach. However, the above-mentioned QTL mapping approaches ignore epistatic SDL and linkage group correction. The two issues need to be addressed in QTL mapping.

SDL epistasis is a type of gene interaction [19]. Törjék et al. [20] indicated that marker segregation distortion is due to reduced fertility caused by epistatic interaction. Recently, some similar results have been reported in Drosophila [21], alfalfa [22] and rice [23]–[25]. Therefore, SDL epistasis should be considered in QTL mapping methodology. Multi-QTL mapping is now the state-of-the-art method. However, it is difficult to implement under the maximum-likelihood framework. At present the Bayesian method implemented via expectation-maximization (EM) algorithm [26] is specialized to handle complicated models and thus it is the ideal tool for mapping multiple QTL in this study. Accordingly, there is a critical need for an in-depth study of the methodology for multi-QTL mapping using epistatic distorted markers.

The purpose of this study was to develop a statistical method for mapping QTL of quantitative traits using epistatic distorted markers. First, marker information was used to detect epistatic SDL using a maximum likelihood approach. The detected SDL parameters were used to correct linkage groups. Second, the information about the detected epistatic SDL along with the corrected linkage groups was incorporated into a multi-QTL mapping approach. Finally, the proposed method was validated by Monte Carlo simulation studies and real data analysis.

## Methods

Based on the corrected genetic groups [11], molecular marker information from all the *n* F_2_ individuals is used to detect epistatic SDL. We hypothesize that marker segregation distortion is subject to the gametic selection.

Two SDL under consideration are linked, with recombination fraction of *r*. The viabilities of male gametes *Ab*, *aB* and *ab* relatives to *AB* are *v*, *u* and *x*, respectively. The expected frequencies 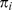 (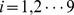) of nine genotypes after gametic selection are listed in **Table 1**.

**Table 1:** Expected frequencies of nine genotypes under gametic selection.

Genotype	Expected frequency	Genotype	Expected frequency
*AABB*	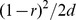	*Aabb*	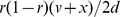
*AABb*	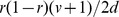	*aaBB*	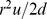
*AAbb*	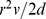	*aaBb*	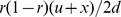
*AaBB*	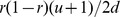	*aabb*	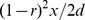
*AaBb*	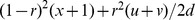		

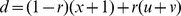

### Mapping Epistatic SDL

Let 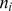 (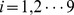) be the observed number of the *i*th genotype for two SDL *A* and *B*, and 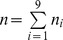 be the total number of individuals. If two linked SDL that exist interaction are resided at marker positions, the marker information is used to detect epistatic SDL. Therefore, the complete data log-likelihood function in F_2_ can be expressed as
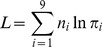
(1)where all the 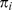 are listed in **Table 1**. Note that *r* in 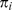 is obtained from the corrected map by Kosambi [27] function. All the SDL parameters *u*, *v* and *x* in 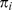 can be estimated by
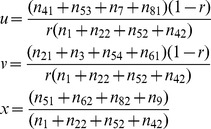
(2)
[11], where 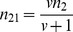, 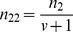, 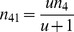, 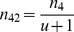, 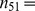 
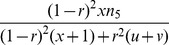
, 
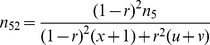
, 
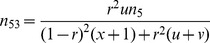
, 
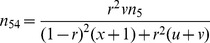
, 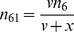, 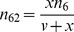, 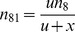 and 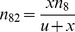. The EM algorithm can be used to estimate all the above parameters [11].

If two linked SDL that exist interaction are not resided at marker positions, the count data 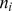 are not observable and we need to substitute them by their expectations. At this case, the EM algorithm is also used to estimate all the above SDL parameters.

In the E-step, the expected numbers of the SDL genotypes can be obtained by the below equations,
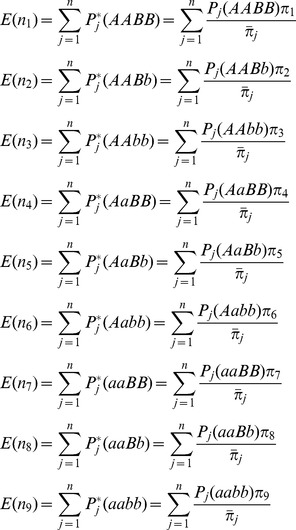
(3)where 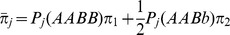 + 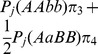 + 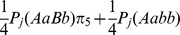 × 
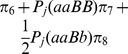
 + 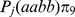; 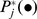 is the posterior probability that incorporates SDL parameters; and 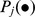 is the probability of the SDL genotypes for individual *j* conditional on marker information. Note that the explanation for the coefficients 1/2 and 1/4 in 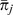 is similar to that in Luo et al. [28]; and 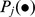 under two SDL is difficult to calculate, fortunately, the method of Kao et al. [29] is available, that is
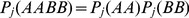
(4)where 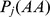 or 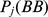 can be calculated from multi-point approach [30].

In the M-step, all the SDL parameters *u*, *v* and *x* can be updated by equations (2).

Repeating E-step and M-step until a certain criterion of convergence is satisfied.

### Interval QTL Mapping using Epistatic Distorted Markers

Let 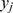 be the observation of quantitative trait for individual *j* in F_2_. For single QTL, the genetic model for quantitative trait may be described as follows
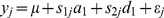
(5)where 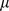 is population mean; 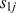 and 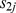 are dummy variables defined as 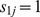 and 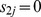 for *QQ*, 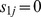 and 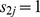 for *Qq*, and 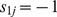 and 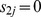 for *qq*, respectively; 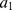 is additive effect; 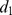 is dominant effect; and 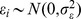.

The posterior probabilities of QTL genotypes conditional on normal markers for the *j*th F_2_ individual are calculated as
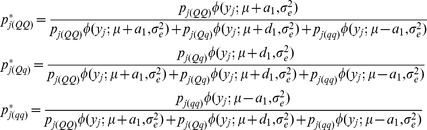
(6)where 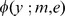 represents normal density function of variable 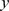 with mean 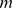 and variance 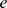; and 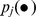 and 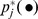 for QTL are similar to 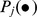 and 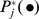 for SDL, respectively.

If epistatic SDL exist, the conditional probabilities 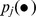 in equation (6) are biased. In this case, the estimates of SDL parameters are used to correct the probabilities. For one locus of epistatic SDL, the expected frequencies of four genotypes are deduced from **Table 1**, and the relative fitness of each genotype is defined as 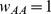, 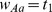, 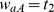 and 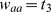. The above two results are listed in **Table 2**. Therefore, their relationship can be found in the below equations
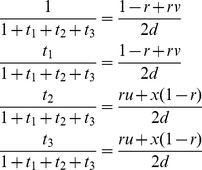
(7)


**Table 2:** Expected frequencies and relative fitness for one locus of epistatic SDL.

Genotype	Expected frequency		Relative fitness
	Before selection	After selection	
AA	1/4	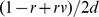	1
Aa	1/4	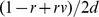	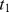
aA	1/4	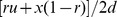	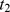
aa	1/4	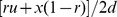	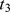

As a result, 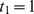 and 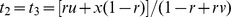, indicating 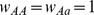 and 
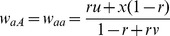
. If the SDL is overlapped with the QTL under study, the above fitnesses are same as those for QTL. Therefore, the conditional probabilities 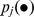 in equation (6) can be corrected using the below equations
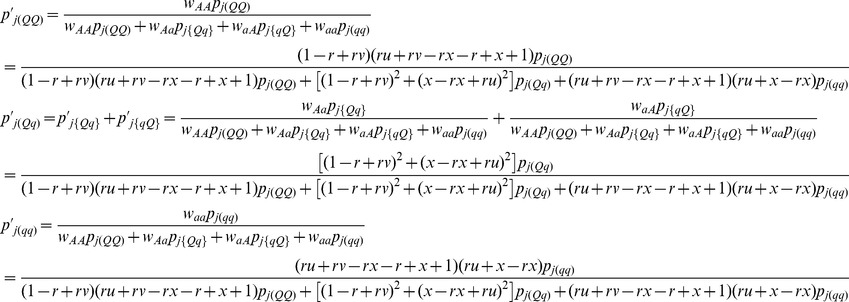
(8)where 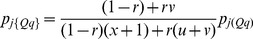 and 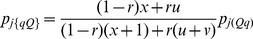.

### Multi-QTL Mapping using Epistatic Distorted Markers

When multiple QTL are taken into account, the model (5) is extended as
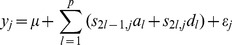
(9)where 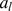 and 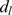 are additive and dominant effects of the *l*th QTL (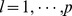), respectively; *p* is the number of putative QTL; and 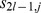 and 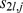 are dummy variables, which are similar to 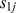 and 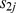 in model (5). This model can be expressed in matrix form
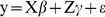
(10)where 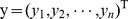; 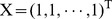; 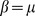;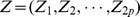; 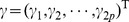; and 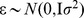.

At present, there are several methods available for estimating the parameters in model (10). There we adopt an empirical Bayes approach [26], and employ normal prior 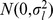 for QTL effect 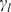 and the scaled inverse 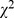 prior 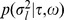 for 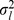, 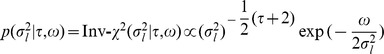, where 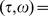 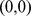 [26]. This procedure for parameter estimation is as follows.

1. Setting initial values: 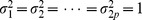, 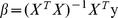, 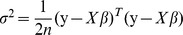.
2. E-step: QTL effect can be predicted by 
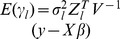
, where 
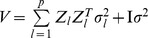
, 
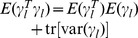
, and 
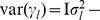
 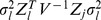.
3. M-step: update parameters 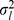, 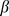 and residual error variance

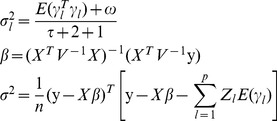
(11)Repeat E-step and M-step until a certain criterion of convergence is satisfied.

In summary, the proposed approach includes three steps. First, marker information is used to detect epistatic SDL. Then, the estimates of SDL parameters are used to correct conditional probabilities of QTL genotypes. Finally, the corrected probabilities are incorporated into a multi-QTL mapping approach.

## Results

### Monte Carlo Simulation Studies

The purpose of the simulation experiment was to evaluate the statistical performance of the proposed approach by changing SDL heritability, QTL heritability, sample size, and genetic distance between SDL and QTL, respectively.

#### Effect of SDL heritability on new method

In the first simulated experiment, the simulated genome consisted of one chromosome, and twenty-one evenly spaced co-dominant markers covered the chromosome with an average marker interval of 5.0 cM. We simulated a single QTL and two SDL, all of which overlapped with markers. The single QTL with 0.10 heritability was located at marker position 25 cM, and two SDL each with heritabilities of 0.05, 0.10 and 0.15 were placed at marker positions 20 cM and 30 cM, respectively. The genetic parameters in F_2_ population with a sample size of 300 were as follows: *a* = *d = *0.3849 (QTL effects), 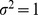, and 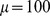. The phenotypic values for quantitative trait are simulated by model (9). The parameters for viability selection were set at *u* = *v* = *x = *0.5141 (5%), 0.3615 (10%) and 0.1617 (15%), respectively. These parameters could be transferred into SDL effects [11]. These SDL genotypic effects along with random error were used to simulate phenotypic values of viability selection. If the value is larger than zero, this individual remains, or it is deleted from the simulated population. Each treatment was replicated 200 times. In the analyses of each simulated dataset, two approaches were adopted: 1) New method, the proposed method in this study; and 2) Old method, the modified method of Wen et al. [10] with linkage groups corrected by epistatic distorted markers. The mean, absolute bias and standard deviation among the estimates obtained from 200 replicates were used to indicate the precision, and paired *t* test was used to compare the above two methods. All simulation parameters were given in **Table 3**.

**Table 3:** Simulated parameters in all the Monte Carlo simulation experiments.

Case	Position (cM)			Distance ofQTL and SDL (cM)	Heritability (%)		Sample size
	SDL_1_	QTL	SDL_2_		SDL_1_ = SDL_2_	QTL	
1	20	25	30	5	5, 10, 15	10	300
2	20	25	30	5	10	5, 10, 15	300
3	20	25	30	5	10	10	100, 200, 300
4	20	22, 25, 30	24, 30, 40	2, 5, 10	15	10	300

**Figure 1:**
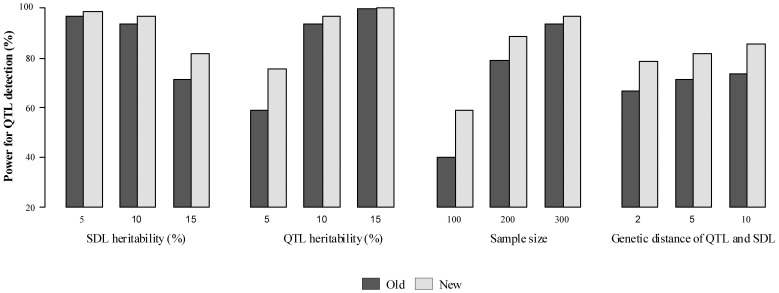
Power of QTL detection under various situations. Old: the modified method of Wen et al. (2013) with the corrected linkage groups; New: the proposed method in this study.

**Figure 2:**
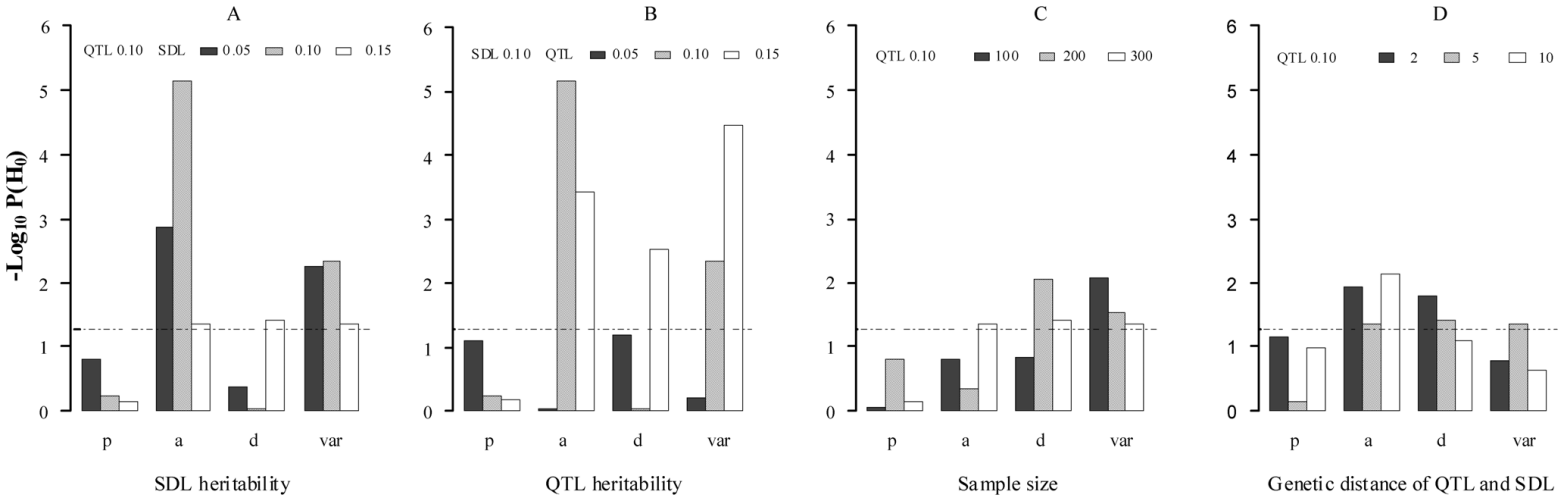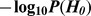 for QTL parameters in the paired *t* test between the old and new methods. *p*: QTL position; *a*: additive effect of QTL; *d*: dominant effect of QTL; *var*: residual variance. The *dashed line* represents the critical value at the 0.05 level of significance.

**Figure 3:**
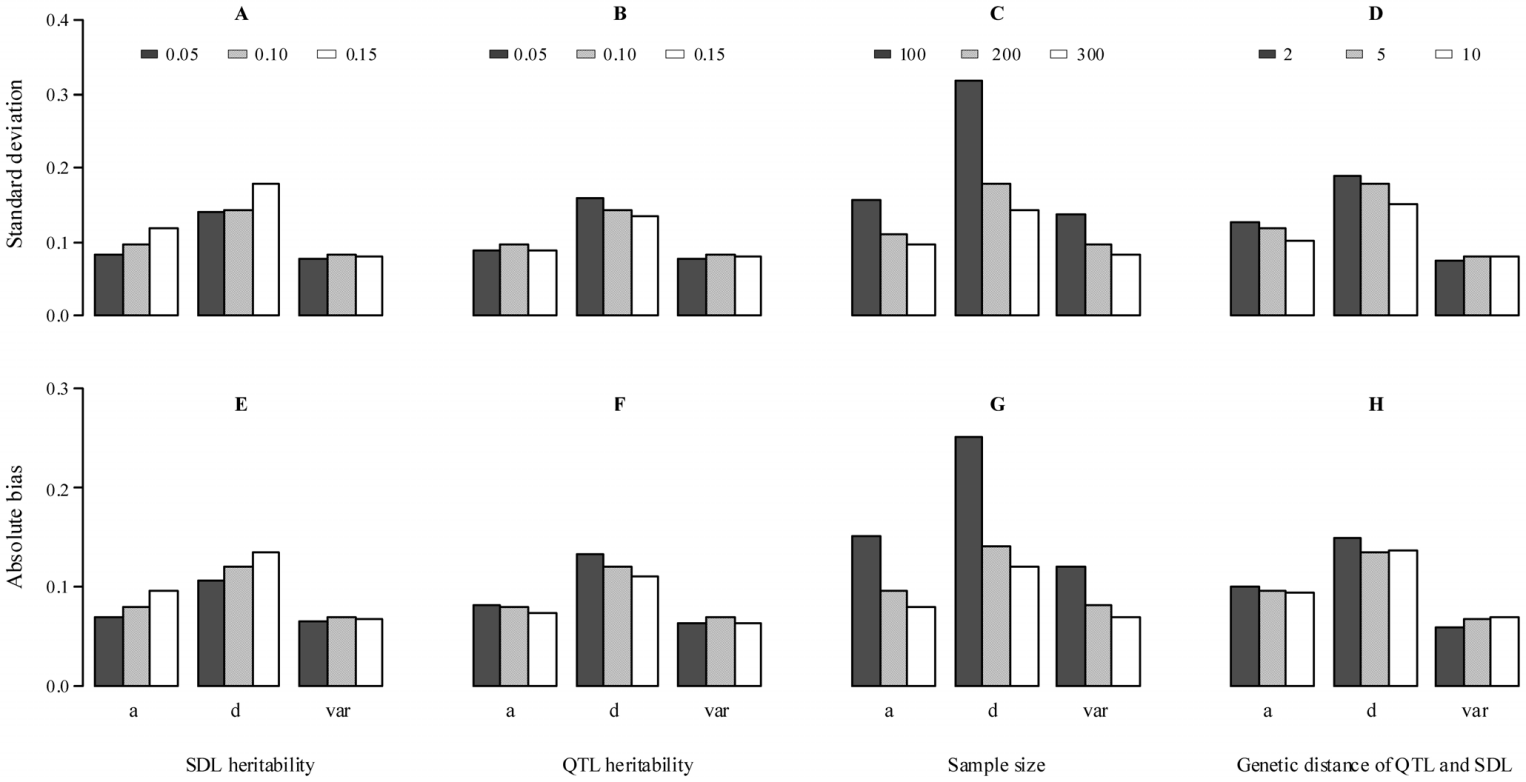
Standard deviation and absolute bias for QTL estimates in the new method. *a*: additive effect; *d*: dominant effect; and *var*: residual variance.

All the results are listed in **Table S1** and **Figs. 1**, **2A** **,** **3A and 3E**. Results showed that the ability to detect QTL decreased as SDL heritability increased in both two methods (**Table S1)**. However, some differences between the two methods were observed if SDL heritability was fixed. For example, the power for QTL detection is higher for new method than for old one (**Fig. 1**); and significant differences for QTL parameter estimates, QTL additive effect and residual variance, exist (**Fig. 2A**). The standard deviations and absolute biases for the estimates of QTL additive and dominance effects are all small, although the accuracy of the new method decreases as SDL heritability increases (**Fig. 3A** and 3E). The unbiasedness is better for the new method than for old one, although the difference of SD between the two methods is not obvious (Table S1).

#### Effect of QTL heritability on new method

In the second simulation experiment, the effect of QTL size on the new method was assessed by setting QTL heritability as 0.05, 0.10 and 0.15, indicating *a* = *d* = 0.2649, 0.3849 and 0.4851, respectively. SDL heritability was set as 0.10. Other parameters were the same as those in the first simulation experiment (**Table 3**). All the results were listed in **Table S2** and **Figs. 1**, **2B** **,** **3B and 3F**. Results showed the similar trends in the first simulated experiment. In addition, the difference of QTL detection power between the above two methods decrease as the increase of QTL heritability (**Fig. 1**), and significant difference for QTL dominant effect exists under QTL heritability of 0.15 (**Fig. 2B**). As for the accuracy of the new method, a general trend is observed (**Figs. 3B and 3F**). In addition, the unbiasedness is also better for the new method than for old one (Table S2).

#### Effect of sample size on new method

The third simulation experiment was designed to investigate the effects of sample size on the new method by setting sample size as 100, 200 and 300. Heritability of each SDL and QTL was set as 0.10. Other parameters were the same as those in the first simulation experiment (**Table 3**). All the results were listed in **Table S3 and** **Figs. 1**, **2C** **,** **3C and 3G**. Results showed a general trend in QTL mapping. In addition, significant differences exist in QTL parameter estimates, i.e., QTL dominant effect and residual variance (**Fig. 2C**). As for the accuracy for parameter estimation, the results are similar to those in the second simulation experiment (**Fig. 3C and 3G**).

#### Effect of genetic distance between QTL and SDL on new method

The last simulation was performed to evaluate the effect of genetic distance between SDL and QTL on the new method by setting the distance as 2, 5 and 10 cM. All the parameters were shown in **Table 3**, and all the results were showed in **Table S4** and **Figs. 1**, **2D** **,** **3D and 3H**. Results showed a general trend in QTL mapping. In addition, significant differences exist in QTL parameter estimates, i.e., QTL additive and dominant effects (**Fig. 2D**). The standard deviations of QTL additive and dominant effects decrease as the genetic distance between QTL and SDL increases (**Fig. 3D**), the absolute bias for QTL additive effect has a same trend as standard deviation (**Fig. 3H**).

### Real Data Analysis

The mouse dataset of 304 F_2_ individuals, derived from MapMaker/Exp v3.0b [31], was used for the demonstration. Twelve RFLP markers were divided into two linkage groups. Each linkage group was corrected by the method of Xie [11], and the corrected length was 132.36 cM. Using the corrected linkage groups, QTL for weight in mouse was detected by three approaches, the above two methods and composite interval mapping [32]. When the marker density is too sparse (>1 cM), a virtual marker (treated as missing data) may be inserted. In the case of incomplete marker information, the corrected conditional probabilities were calculated by equation (8) and these probabilities were used to calculate dummy variables for all putative QTL, i.e., 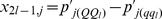 and 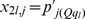. The LOD threshold was set at 3.0 for QTL detection.

**Figure 4:**
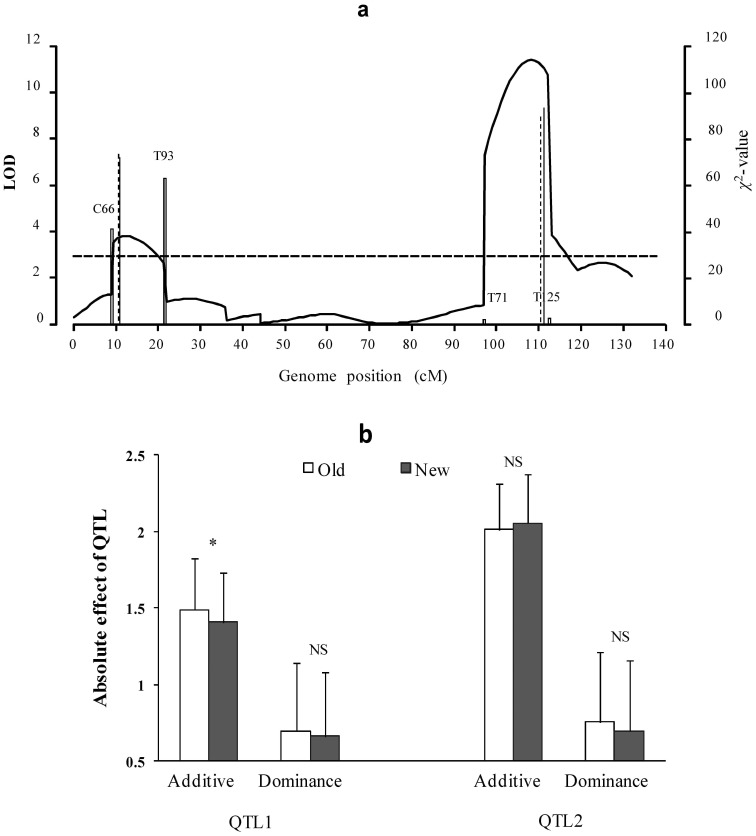
Mapping QTL for weight in 333 mouse F_2_ individuals. (a) LOD scores using composite interval mapping (CIM, *curve*), old (*solid vertical line*) and new (*dashed vertical line*) methods. *Dashed horizontal line* represents critical value for significant QTL. *Hollow vertical line* indicates 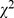 value of segregation distortion test for marker interval of QTL; (b) QTL absolute effects, detected by old and new methods. NS and *star* indicate no difference and significant difference at the 0.01 level between old and new methods, respectively.

All the results are listed in **Table S5** and shown in **Fig. 4**. Among two QTL detected by all the three approaches, no significant difference for estimates of the second QTL is observed between the old and new methods (**Fig. 4b**). However, significant difference for additive effect of the first QTL is found (**Fig. 4b**). This is because that the first QTL is located between epistatic distorted markers C66 and T93 (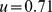, 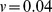, 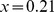, 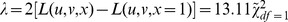, 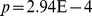), and the second QTL is located at marker region with normal segregation. The result further confirms the conclusions of the simulation studies.

## Discussion

Marker segregation distortion is a common phenomenon observed in QTL analysis [6], thus Wen et al. [10] proposed multi-QTL mapping using distorted markers. However, this work needs to be addressed in two aspects. First, linkage groups in Wen et al. [10] are not corrected by making use of distorted marker information. Second, epistasis between two SDL is not considered although the epistasis is very important. To overcome the above shortcomings, a new approach was proposed in this study. In the simulated data analyses, some new results were found, for example, QTL detection power is slightly higher for the new method than for the above old method and significant difference for dominant effect of QTL is observed between the above two methods (Fig. 2). In real data analyses, epistatic distorted makers were detected. Therefore, the new approach is valuable. To further validate the new method, we assume no epistasis between two linked SDL. Results from a Monte Carlo simulation experiment show that similar results for QTL detection power and parameter estimates between the old and new methods were observed (Table S7), indicating that the new method works well.

In this study new method is based on gametic selection. This is because gametic selection caused by epistatic SDL is often reported [21], [23]–[24], [33]. In this case, multi-QTL mapping approach can be set up by incorporating viability coefficients of male gametes (*u*, *v* and *x*) into QTL mapping approach. Note that the fitness model can be linked with the quantitative genetics model for viability selection [11], [28]. Therefore, epistatic SDL effect and size can be easily calculated. As for zygotic selection, once epistatic SDL is identified by the method of Xie [11], the epistatic SDL information can be used to correct conditional probabilities of QTL genotypes in multi-QTL mapping approach. Therefore, multi-QTL mapping approach under zygotic selection can be easily set up.

In real data analysis, corrected linkage groups are also useful. To explain this issue, we re-analyze real dataset in Wen et al. [10]. As compared with the results from Wen et al. [10], one additional QTL was further detected by the old and new methods (**Table S6**), indicating that the corrected linkage groups using distorted markers can increase the power of QTL mapping. This result is consistent with that in Monte Carlo simulation studies in this study. As for the same QTL detected by the old and new methods, no significant differences are identified. This is because no epistasis SDL was mapped. As for the third QTL, the position from Wen et al. [10] is different from those from the old and new methods; and as for the fourth QTL, the additive effect estimate from Wen et al. [10] is different from those from the old and new methods. The possible reason is derived from the corrected linkage groups.

This method may be extended to the QTL-by-QTL interaction detection and additional bi-parental populations, e.g. backcross, recombination inbred lines and doubled haploid lines. Although co-dominant markers were adopted in this study, missing and dominant markers are also available, as shown in the real data analysis in this study. The related results can be found in Xie [11]. If more than two distorted markers exist in a same linkage group, a two-dimensional scanning approach in this study was also used to detect the epistasis. The source codes for R program and the much more user-friendly interface software will be available soon.

## Supporting Information

Table S1 **Effect of SDL heritability on new method.**
(DOC)

Table S2 **Effect of QTL heritability on new method.**
(DOC)

Table S3 **Effect of sample size on new method.**
(DOC)

Table S4 **Effect of genetic distance between QTL and SDL on new method.**
(DOC)

Table S5 **Mapping QTL for weight in 333 mouse F_2_ individuals using composite interval mapping (CIM), old and new methods.**
(DOC)

Table S6 **Mapping QTL for dried soymilk in 222 soybean F_2∶4_ families using Wen et al. (2013), old and new methods.**
(DOC)

Table S7 **Estimates of SDL and QTL parameters by old and new methods at the case that epistasis between two linked SDL is absent.**
(DOC)
