# Supplementary material for: Multi-QTL Mapping for Quantitative Traits Using Epistatic Distorted Markers
Source: PLoS One. 2013 Jul 9;8(7):e68510. doi: 10.1371/journal.pone.0068510 (PMC3706401; doi:10.1371/journal.pone.0068510)
Supplement: Table S1 — Effect of SDL heritability on new method. (DOC) [file pone.0068510.s001.doc]

**Table S1.** Effect of SDL heritability on new method (QTL heritability: 10%, sample size: 300)

| SDL Heritability  (%) | SDL | | | | | Method | QTL | | | | |
| --- | --- | --- | --- | --- | --- | --- | --- | --- | --- | --- | --- |
|  | Power (%) | Position | *u* | *v* | *x* |  | Power (%) | Position | *a* | *d* | *σ*^2^ |
| 5 | 92 | 18.98/31.10  (7.14/11.79) | 0.4828  (0.2713) | 0.5026  (0.2633) | 0.5016  (0.0782) | Old | 96.5 | 26.08  (7.97) | 0.4036  (0.0796) | 0.3898  (0.1402) | 0.9784  (0.0774) |
|  |  |  |  |  |  | New | 98.5 | 26.03  (7.92) | 0.3984  (0.0827) | 0.3865  (0.1401) | 0.9796  (0.0778) |
| 10 | 100 | 20.98/28.37  (5.11/6.38) | 0.3477  (0.2072) | 0.3430  (0.1872) | 0.3627  (0.0681) | Old | 93.5 | 24.88  (6.79) | 0.4183  (0.0944) | 0.4218  (0.1437) | 0.9719  (0.0850) |
|  |  |  |  |  |  | New | 96.5 | 24.70  (6.94) | 0.4122  (0.0974) | 0.4174  (0.1433) | 0.9736  (0.0847) |
| 15 | 100 | 21.94/27.59  (4.18/4.43) | 0.1808  (0.1436) | 0.1522  (0.1230) | 0.1590  (0.0354) | Old | 71.5 | 27.04  (12.08) | 0.4425  (0.1181) | 0.4411  (0.1879) | 0.9705  (0.0776) |
|  |  |  |  |  |  | New | 81.5 | 27.19  (13.59) | 0.4270  (0.1195) | 0.4274  (0.1803) | 0.9794  (0.0812) |

The standard deviations among 200 replicated simulations are in parentheses.
