# Supplementary material for: Multi-QTL Mapping for Quantitative Traits Using Epistatic Distorted Markers
Source: PLoS One. 2013 Jul 9;8(7):e68510. doi: 10.1371/journal.pone.0068510 (PMC3706401; doi:10.1371/journal.pone.0068510)
Supplement: Table S2 — Effect of QTL heritability on new method. (DOC) [file pone.0068510.s002.doc]

**Table S2. Effect of QTL heritability on new method (SDL heritability: 10%, sample size: 300)**

| QTL Heritability  (%) | SDL | | | | | Method | QTL | | | | |
| --- | --- | --- | --- | --- | --- | --- | --- | --- | --- | --- | --- |
|  | Power (%) | Position | *u* | *v* | *x* |  | Power (%) | Position | *a* | *d* | *σ*^2^ |
| 5 | 100 | 20.24/28.20  (6.07/6.68) | 0.3455  (0.1933) | 0.3427  (0.2070) | 0.3709  (0.0693) | Old | 59 | 25.64  (11.71) | 0.3383  (0.0879) | 0.3173  (0.1716) | 0.9771  (0.0762) |
|  |  |  |  |  |  | New | 75.5 | 26.53  (14.54) | 0.3185  (0.0909) | 0.3065  (0.1598) | 0.9875  (0.0777) |
| 10 | 100 | 20.98/28.37  (5.11/6.38) | 0.3477  (0.2072) | 0.3430  (0.1872) | 0.3627  (0.0681) | Old | 93.5 | 24.88  (6.79) | 0.4183  (0.0944) | 0.4218  (0.1437) | 0.9719  (0.0850) |
|  |  |  |  |  |  | New | 96.5 | 24.70  (6.94) | 0.4122  (0.0974) | 0.4174  (0.1433) | 0.9736  (0.0847) |
| 15 | 100 | 21.50/28.72  (4.86/5.88) | 0.3428  (0.2077) | 0.3655  (0.2031) | 0.3566  (0.0650) | Old | 99.5 | 25.18  (5.53) | 0.5040  (0.0890) | 0.4908  (0.1348) | 0.9964  (0.0794) |
|  |  |  |  |  |  | New | 100 | 25.17  (5.54) | 0.5018  (0.0887) | 0.4878  (0.1360) | 0.9976  (0.0799) |
