# Supplementary material for: Multi-QTL Mapping for Quantitative Traits Using Epistatic Distorted Markers
Source: PLoS One. 2013 Jul 9;8(7):e68510. doi: 10.1371/journal.pone.0068510 (PMC3706401; doi:10.1371/journal.pone.0068510)
Supplement: Table S3 — Effect of sample size on new method. (DOC) [file pone.0068510.s003.doc]

**Table S3.** Effect of sample size on new method (SDL and QTL heritability: 10%)

| Sample size | SDL | | | | | Method | QTL | | | | |
| --- | --- | --- | --- | --- | --- | --- | --- | --- | --- | --- | --- |
|  | Power (%) | Position | *u* | *v* | *x* |  | Power (%) | Position | *a* | *d* | *σ*^2^ |
| 100 | 81 | 18.66/31.25  (7.81/11.14) | 0.3053  (0.2906) | 0.3405  (0.3307) | 0.3242  (0.0990) | Old | 40 | 26.76  (13.49) | 0.5293  (0.1528) | 0.4918  (0.3283) | 0.9060  (0.1304) |
|  |  |  |  |  |  | New | 59 | 27.08  (15.92) | 0.4940  (0.1556) | 0.4685  (0.3171) | 0.9490  (0.1390) |
| 200 | 99 | 20.34/28.70  (6.68/7.61) | 0.3811  (0.2507) | 0.3757  (0.2366) | 0.3660  (0.0809) | Old | 79 | 25.17  (10.15) | 0.4477  (0.1062) | 0.4541  (0.1655) | 0.9713  (0.0981) |
|  |  |  |  |  |  | New | 88.5 | 24.83  (10.37) | 0.4331  (0.1110) | 0.4342  (0.1785) | 0.9763  (0.0968) |
| 300 | 100 | 20.98/28.37  (5.11/6.38) | 0.3477  (0.2072) | 0.3430  (0.1872) | 0.3627  (0.0681) | Old | 93.5 | 24.88  (6.79) | 0.4183  (0.0944) | 0.4218  (0.1437) | 0.9719  (0.0850) |
|  |  |  |  |  |  | New | 96.5 | 24.70  (6.94) | 0.4122  (0.0974) | 0.4174  (0.1433) | 0.9736  (0.0847) |
