# Supplementary material for: Multi-QTL Mapping for Quantitative Traits Using Epistatic Distorted Markers
Source: PLoS One. 2013 Jul 9;8(7):e68510. doi: 10.1371/journal.pone.0068510 (PMC3706401; doi:10.1371/journal.pone.0068510)
Supplement: Table S4 — Effect of genetic distance between QTL and SDL on new method. (DOC) [file pone.0068510.s004.doc]

**Table S4.** Effect of genetic distance between QTL and SDL on new method (SDL heritability: 15%, QTL heritability: 10%)

| Distance of  SDL and QTL (cM) | SDL | | | | | Method | QTL | | | | |
| --- | --- | --- | --- | --- | --- | --- | --- | --- | --- | --- | --- |
|  | Power (%) | Position | *u* | *v* | *x* |  | Power (%) | Position | *a* | *d* | *σ*^2^ |
| 2 | 100 | 20.46/23.02  (3.44/3.71) | 0.1822  (0.1943) | 0.1782  (0.1938) | 0.1569  (0.0387) | Old | 66.5 | 24.55  (14.02) | 0.4596  (0.1093) | 0.4690  (0.1694) | 0.9797  (0.0765) |
|  |  |  |  |  |  | New | 78.5 | 24.61  (15.19) | 0.4315  (0.1278) | 0.4352  (0.1914) | 0.9856  (0.0745) |
| 5 | 100 | 21.94/27.59  (4.18/4.43) | 0.1808  (0.1436) | 0.1522  (0.1230) | 0.1590  (0.0354) | Old | 71.5 | 27.04  (12.08) | 0.4425  (0.1181) | 0.4411  (0.1879) | 0.9705  (0.0776) |
|  |  |  |  |  |  | New | 81.5 | 27.19  (13.59) | 0.4270  (0.1195) | 0.4274  (0.1803) | 0.9794  (0.0812) |
| 10 | 100 | 22.87/34.70  (4.88/5.71) | 0.1403  (0.0935) | 0.1367  (0.0884) | 0.1621  (0.0421) | Old | 73.5 | 30.14  (10.61) | 0.4562  (0.1055) | 0.4658  (0.1540) | 0.9726  (0.0784) |
|  |  |  |  |  |  | New | 85.5 | 29.85  (10.24) | 0.4425  (0.1042) | 0.4493  (0.1527) | 0.9823  (0.0812) |
