# Supplementary material for: Multi-QTL Mapping for Quantitative Traits Using Epistatic Distorted Markers
Source: PLoS One. 2013 Jul 9;8(7):e68510. doi: 10.1371/journal.pone.0068510 (PMC3706401; doi:10.1371/journal.pone.0068510)
Supplement: Table S5 — Mapping QTL for weight in 333 mouse F2 individuals using composite interval mapping (CIM), old and new methods. (DOC) [file pone.0068510.s005.doc]

**Table S5.** Mapping QTL for weight in 333 mouse F_2_ individuals using composite interval mapping (CIM), old and new methods

| QTL | Method | Interval | | | | Linkage | Position |  | Additive | |  | Dominance | | LOD | *h*^2^(%) |
| --- | --- | --- | --- | --- | --- | --- | --- | --- | --- | --- | --- | --- | --- | --- | --- |
|  |  | Marker1 | $\chi^{2}$ | Marker2 | $\chi^{2}$ |  |  |  | Estimate | ­*P*(H_0_) |  | Estimate | *P*(H_0_) |  |  |
| 1 | CIM | C66 | 40.30** | T93 | 65.99** | 1 | 12.40 |  | 1.72 |  |  | -1.02 |  | 3.84 | 12.39 |
|  | Old |  |  |  |  |  | 10.34 |  | 1.49(0.34) | 0.002 |  | -0.70(0.44) | 0.32 | 7.43 | 9.09 |
|  | New |  |  |  |  |  | 9.39 |  | 1.41(0.32) |  |  | -0.66(0.41) |  | 6.90 | 8.21 |
| 2 | CIM | T71 | 1.06 | T125 | 3.41 | 2 | 108.27 |  | 2.61 |  |  | -0.86 |  | 11.42 | 22.60 |
|  | Old |  |  |  |  |  | 104.22 |  | 2.01(0.30) | 0.09 |  | -0.76(0.45) | 0.10 | 9.93 | 14.98 |
|  | New |  |  |  |  |  | 105.25 |  | 2.06(0.32) |  |  | -0.70(0.46) |  | 9.36 | 15.37 |

The standard deviations of additive and dominance effect were in parentheses; **: at the 0.01 level of significance.
