# Supplementary material for: Multi-QTL Mapping for Quantitative Traits Using Epistatic Distorted Markers
Source: PLoS One. 2013 Jul 9;8(7):e68510. doi: 10.1371/journal.pone.0068510 (PMC3706401; doi:10.1371/journal.pone.0068510)
Supplement: Table S6 — Mapping QTL for dried soymilk in 222 soybean F2∶4 families using Wen et al. (2013), old and new methods. (DOC) [file pone.0068510.s006.doc]

**Table S6.** Mapping QTL for dried soymilk in 222 soybean F_2:4_ families using Wen et al.(2013), old and new methods

| QTL | Method | Interval | | | | Chromosome | Position | |  | Additive | |  | Dominance | | LOD |
| --- | --- | --- | --- | --- | --- | --- | --- | --- | --- | --- | --- | --- | --- | --- | --- |
|  |  | Marker1 | $\chi^{2}$ | Marker2 | $\chi^{2}$ |  | Estimate | *p* ^+^ |  | Estimate | *p* |  | Estimate | *p* |  |
| 1 | Wen et al. [10] | sat_344 | 3.25 | satt449 | 2.38 | 5(A1) | 36.04(4.77) |  |  | 2.27(0.32) |  |  | 0.78(0.94) |  | 3.19 |
|  | Old |  |  |  |  |  | 35.20(4.52) | 0.83 |  | 2.19(0.18) | 0.26 |  | 0.99(1.00) | 0.30 | 3.16 |
|  | New |  |  |  |  |  | 35.56(4.78) |  |  | 2.00(0.68) |  |  | 1.39(1.19) |  | 3.84 |
| 2 | Wen et al. [10] | satt449 | 2.38 | sat_356 | 0.41 | 5(A1) | 53.37(5.09) |  |  | 2.19(0.81) |  |  | 1.29(1.03) |  | 3.32 |
|  | Old |  |  |  |  |  | 53.22(3.79) | 0.70 |  | 2.33 (0.37) | 0.24 |  | 0.96(1.27) | 0.59 | 3.56 |
|  | New |  |  |  |  |  | 53.66(4.04) |  |  | 2.17(0.54) |  |  | 1.15(1.18) |  | 4.08 |
| 3 | Wen et al. [10] | satt355 | 23.85** | satt070 | 0.51 | 14(B2) | 152.48(7.83) |  |  | 2.24(0.33) |  |  | -0.75(0.90) |  | 3.33 |
|  | Old |  |  |  |  |  | 134.13(5.12) | 0.15 |  | 2.24(0.26) | 0.31 |  | -0.52(0.90) | 0.36 | 3.51 |
|  | New |  |  |  |  |  | 128.88(9.33) |  |  | 2.03(0.55) |  |  | 0.27(2.45) |  | 3.78 |
| 4 | Wen et al. [10] | satt632 | 107.28** | sat_228 | 22.73** | 16(J) | 1540.09(12.63) |  |  | -0.66(0.85) |  |  | -3.61(0.76) |  | 3.23 |
|  | Old |  |  |  |  |  | 1539.75(1.38) | 0.69 |  | 0.06(0.27) | 0.80 |  | -3.54(0.22) | 0.81 | 2.94 |
|  | New |  |  |  |  |  | 1539.40(1.51) |  |  | 0.10(0.27) |  |  | -3.50(0.21) |  | 5.19 |
| 5 | Old | sat_418 | 2.23 | sat_419 | 6.84* | 20(I) | 882.07(3.82) | 0.10 |  | -0.84(0.15) | 0.57 |  | 3.56(0.40) | 0.58 | 3.36 |
|  | New |  |  |  |  |  | 878.88(5.36) |  |  | -0.79(0.19) |  |  | 3.48(0.23) |  | 5.54 |

^+^：The t-test for QTL parameters was conducted between old and new methods; * and **: at the 0.05 and 0.01 level of significance.
