# Supplementary material for: Multi-QTL Mapping for Quantitative Traits Using Epistatic Distorted Markers
Source: PLoS One. 2013 Jul 9;8(7):e68510. doi: 10.1371/journal.pone.0068510 (PMC3706401; doi:10.1371/journal.pone.0068510)
Supplement: Table S7 — Estimates of SDL and QTL parameters by old and new methods at the case that epistasis between two linked SDL is absent. (DOC) [file pone.0068510.s007.doc]

**Table S7.** Estimates of SDL and QTL parameters by old and new methods at the case that epistasis between two linked SDL is absent

| SDL | | | | | Method | QTL | | | | |
| --- | --- | --- | --- | --- | --- | --- | --- | --- | --- | --- |
| Power (%) | Position | *u* | *v* | *x* |  | Power (%) | Position | *a* | *d* | *σ*^2^ |
| 78.5 | 20.99/27.15  (2.42/2.60) | 0.1216  (0.1817) | 0.1349  (0.1784) | 1.0037  (0.11904) | Old | 99 | 24.46  (4.31) | 0.3959  (0.0852) | 0.3918  (0.1261) | 0.9862  (0.0788) |
|  |  |  |  |  | New | 99.5 | 24.65  (4.69) | 0.3944  (0.0867) | 0.3906  (0.1260) | 0.9871  (0.0792) |

SDL heritability: 15%; QTL heritability: 10%; and sample size: 300.
